# Shortfalls in home quarantine for COVID-19 prevention: a case report

**DOI:** 10.11604/pamj.supp.2020.37.1.27058

**Published:** 2020-11-21

**Authors:** Chidzani Catherine Mbenge, Lebapotswe Tlale, Lisani Ntoni, Naledi Mokgethi, Sidney Otladisa Kololo, Rinett Pharatlhatlhe, Tuduetso Molefi

**Affiliations:** 1 University of Botswana, 4775 Notwane Road, Gaborone, Botswana,; 2 Ministry of Health and Wellness, Private Bag 0038, Gaborone, Botswana

**Keywords:** COVID-19, home quarantine, local transmission, case report

## Abstract

Quarantine of COVID-19 high risk groups is one strategy of interrupting chains of transmissions of the disease. We describe a COVID-19 imported case placed under home quarantine that resulted in 8 local transmission cases at the beginning of the COVID-19 epidemic in Botswana. This case report highlights that home quarantine if not well managed can lead to a surge in local cases and drawing from the literature we propose recommendations of strengthening home quarantine.

## Introduction

Severe Acute Respiratory Syndrome Coronavirus 2 (SARS-COV-2) is a novel coronavirus that was initially detected in December 2019 when clusters of patients presented with unusual patterns of pneumonia in Wuhan, China [1]. From China there was evident human to human transmission and the resultant importation of the virus internationally leading to a global pandemic. Countries had to implement quarantine measures for returning travelers from highly affected countries and contacts of confirmed cases in order to stop local transmissions [2]. The World Health Organization describes quarantine as separation of asymptomatic people exposed to an infectious disease from the community with the mandate of monitoring symptom development for early detection of cases and cutting down chains of transmissions [2]. It can be carried out either at homes or in facilities. During SARS 2003 outbreak, countries like Singapore and Taiwan successfully managed the disease through efficient home quarantines [3,4].

From the 30^th^ of January 2020, Botswana instituted points of entry screening for COVID-19 at airports and land crossings. Travelers from highly affected countries were screened and asymptomatic ones were advised to go on 14 days home quarantine up until the 24^th^ of March 2020 when the country put in place facility quarantine. However there was inadequate active follow up of clients on home quarantine. As of 31^st^ May 2020, Botswana had 38 confirmed cases, with only 11 local transmissions of which 8 were linked to one imported case (unpublished data). The number of local transmissions remained the same until cluster outbreaks were detected in July 2020. We seek to describe an imported case of COVID-19 that breached home quarantine regulations and the subsequent development of local transmissions in order to come up with recommendations to strengthen home quarantine strategy.

## Patient and Observation

A young male, XY, returned to Botswana from the United Kingdom on the 22^nd^ of March 2020. He was screened at Botswana's largest air point of entry, Sir Seretse Khama International Airport, using a screening questionnaire and temperature check. He was found to be asymptomatic and advised to go under home quarantine. As he did not have his own transport from the airport he was picked up by a group of friends who then held a welcome home session for him at a residence in Gaborone. After 2 days XY travelled to a village 50km from Gaborone where he quarantined with his friend, XX, without reporting to the local health team. However, he then developed flu-like symptoms on the 28^th^ of March 2020 and called the local health team who upon learning that he arrived from a COVID-19 highly affected country, came prepared in full personal protective equipment and admitted him to the local isolation center. His friend was also put into isolation the following day. They both tested positive for SARS-COV-2 by polymerase chain reaction (PCR) and were Botswana's COVID-19 cases number 5 and 6 and his friend was the first local transmission case in Botswana. XY and XX contacts were all line listed and all close contacts were home quarantined and tested. 2 other XY's friends who worked at the same workplace of 105 employees became confirmed cases on the 8^th^ of April 2020. The employees and immediate families of the 2 friends were quarantined and tested where 5 more employees became confirmed cases. No further cases were discovered from their contacts.

## Discussion

The breach in home quarantine regulations led to the initial 8 local transmissions in Botswana. The shortfalls identified from this case were that the index was given verbal instructions at the airport, had improper transport arrangement, mingled with many people and there was no active follow up in quarantine. It is our argument that this was a preventable situation had there been proper quarantine measures in place. As countries like Botswana are considering lifting travel bans and resuming international travel amidst the COVID-19 pandemic, quarantine of travelers from highly affected countries might still be necessary. It is not only returning travelers and close contacts of cases that are monitored at home but confirmed COVID-19 cases not requiring hospitalization can be placed under home isolation. Best practices should be in place so that these high risk groups monitored at home do not spread the COVID-19 infection defeating countries' efforts of epidemic control. Some factors associated with adherence to quarantine include perceived risk of disease, perceived benefit of quarantine, shortage of essential supplies and financial implications [5]. Therefore, whenever home quarantine is utilized it needs to be well organized and highly monitored to be effective [3,4,6].

Legally binding agreements signed by clients going under quarantine are one way of ensuring compliance. During the SARS outbreak in 2003 Singapore ensured compliance to home quarantine by serving clients with the Home Quarantine Orders (HQO) and fining those who breach it in addition to surveillance, enforcement, health education, transport, and financial support to those losing income by being in quarantine. Only 26 out of 7863 contacts of SARS cases served with an HQO breached the rules and were penalized [3]. Similarly, during the COVID-19 outbreak, Singapore instituted the Stay at Home Notice (SAH) for returning travelers that is as legally enforceable as the HQO to ensure compliance [7]. Taiwan has also previously achieved the high compliance rate to quarantine through registration, active monitoring, video monitoring and fines [4]. Through the Public Health Act, Botswana should legally bind those undergoing quarantine and rule breakers be fined as specified in the Act [8]. Additionally, all those who are caught breaking home quarantine rules should be moved to facility quarantine.

Community structures can be mobilized to support home quarantine. An example is Shenzhen, China that controlled COVID-19 transmissions through effective management of home quarantine using the “Three in One” Task Force comprised of community work stations, community health centers and community police where community workers ordered and delivered necessities for those in quarantine and actively monitored them twice a day. This organized home quarantine had good results as only three out of 2,004 persons on home quarantine had confirmed COVID-19 infection [9]. African countries like Botswana can also use community structures such as village leadership, village development committees and community volunteers to monitor compliance of people on home quarantine. The neighborhood watch program (community policing) successfully used against crime can be extended to report neighbors breaking quarantine rules. High volumes of people on home quarantine require a lot of manpower to follow up clients daily. However, in the case of Singapore, they outsourced the service to a security agency and used auxiliary police officers instead of health officers in serving the HQO at homes. The officers made random checks by calling the clients twice daily to ensure that they stay home [3]. This is a stringent measure that can be adopted by countries to ensure compliance.

Brooks *et al*. emphasized the need for psychosocial and other support services to minimize the stress of quarantine on individuals and ensure compliance [10]. The following is useful: information to help people understand why they need to quarantine and provision of food, general and medical supplies [5]. It is equally important that home assessments are carried out to ensure that conditions at home are favorable for home quarantine [2]. Technological advances can be adopted to instill compliance. In Shenzhen, there was use of a smartphone application called Ishenzhen which coded high risk and non-high risk groups and upon inspection only non-high risk people were allowed to go into the community [9]. Singapore tightened surveillance through camera installation at homes that mandated the client to present himself in front of the camera when called to prove that they are home [3]. Similarly, Taiwan also resorted to video surveillance of people who breached quarantine rules [4]. Ethical considerations should be taken not to step on the rights of individuals whenever technology is employed, for example in the use of electronic bracelets to track individuals on quarantine.

**Ethics and consent:** this is work of the operational Botswana´s COVID-19 national contact tracing subcommittee that produces case reports as part of contact tracing. Permission to publish was sought and granted by the ethics body under Ministry of Health and Wellness Office of Health Research and Development. The case study uses existing contact tracing data reports and upheld confidentiality of patient´s information.

## Conclusion

Well organized home quarantines are a feasible alternative to facility quarantine for the control of communicable diseases like COVID-19. If not well managed, breaches in home quarantines can lead to the spread of local transmissions in the community. Home quarantine should be reinforced through legal binding quarantine orders, home assessments, health education, use of community structures, provision of support services in quarantines, use of technology for surveillance and taking legal action against those who break rules.
